# High-Fat Diet Induces Neuroinflammation and Mitochondrial Impairment in Mice Cerebral Cortex and Synaptic Fraction

**DOI:** 10.3389/fncel.2019.00509

**Published:** 2019-11-12

**Authors:** Gina Cavaliere, Giovanna Trinchese, Eduardo Penna, Fabiano Cimmino, Claudio Pirozzi, Adriano Lama, Chiara Annunziata, Angela Catapano, Giuseppina Mattace Raso, Rosaria Meli, Marcellino Monda, Giovanni Messina, Christian Zammit, Marianna Crispino, Maria Pina Mollica

**Affiliations:** ^1^Department of Biology, University of Naples Federico II, Naples, Italy; ^2^Department of Pharmacy, University of Naples Federico II, Naples, Italy; ^3^Unit of Dietetics and Sports Medicine, Section of Human Physiology, Department of Experimental Medicine, University of Campania Luigi Vanvitelli, Naples, Italy; ^4^Department of Clinical and Experimental Medicine, University of Foggia, Foggia, Italy; ^5^Department of Anatomy, Faculty of Medicine and Surgery, University of Malta, Msida, Malta

**Keywords:** high-fat diet, neuroinflammation, mitochondria, synaptic plasticity, BDNF

## Abstract

Brain mitochondrial dysfunction is involved in the development of neurological and neurodegenerative diseases. Mitochondria specifically located at synapses play a key role in providing energy to support synaptic functions and plasticity, thus their defects may lead to synaptic failure, which is a common hallmark of neurodegenerative diseases. High-Fat Diet (HFD) consumption increases brain oxidative stress and impairs brain mitochondrial functions, although the underlying mechanisms are not completely understood. The aim of our study is to analyze neuroinflammation and mitochondrial dysfunctions in brain cortex and synaptosomal fraction isolated from a mouse model of diet-induced obesity. Male C57Bl/6 mice were divided into two groups fed a standard diet or HFD for 18 weeks. At the end of the treatment, inflammation (detected by ELISA), antioxidant state (measured by enzymatic activity), mitochondrial functions and efficiency (detected by oxidative capacity and Seahorse analysis), and brain-derived neurotrophic factor (BDNF) pathway (analyzed by western blot) were determined in brain cortex and synaptosomal fraction. In HFD animals, we observed an increase in inflammatory parameters and oxidative stress and a decrease in mitochondrial oxidative capacity both in the brain cortex and synaptosomal fraction. These alterations parallel with modulation of BDNF, a brain key signaling molecule that is linking synaptic plasticity and energy metabolism. Neuroinflammation HFD-dependent negatively affects BDNF pathway and mitochondrial activity in the brain cortex. The effect is even more pronounced in the synaptic region, where the impaired energy supply may have a negative impact on neuronal plasticity.

## Introduction

High-Fat diet (HFD) consumption induces obesity-related metabolic disorders characterized by low-grade inflammation (Mollica et al., 2011). Inflammation is a response to stress designed for adaptation and recovery from insults (Hernández-Aguilera et al., 2013). When an inflammatory state is prolonged, it can compromise cell functions, leading to altered metabolism and associated pathological conditions (Hernández-Aguilera et al., 2013). Mitochondria, the primary cellular energy-generating system, produce key factors during inflammation and oxidation and constitute the main source of reactive oxygen species (ROS). Therefore, mitochondrial dysfunction is related to inflammation and other energy-dependent disturbances, where the generation of ROS exceeds the physiological antioxidant protective activity, causing cellular oxidative damage (Chan, 2006). In the brain, neuroinflammation is an important risk factor for neurodegenerative disorders, characterized by learning and cognitive decline that depends on altered neuronal connectivity and atypical synaptic plasticity (Kumar, 2018). Numerous data indicate that HFD may activate signaling pathways with deleterious effects in several brain regions such as the hippocampus and brain cortex (Dutheil et al., 2016; Chen et al., 2019). The non-coding RNAs are assuming escalating importance in the regulation of metabolic disease at RNA levels, linking nutritional influence, neuroinflammation and brain-derived neurotrophic factor (BDNF) pathway (Hanin et al., 2018; Haviv et al., 2018; Meydan et al., 2018). BDNF is a neurotrophin playing a key role in the physiology and pathology of the brain (Lima Giacobbo et al., 2019). In particular, neuroinflammation HFD-induced is known to affect BDNF-related pathways in several brain regions (Mi et al., 2017; Zhao et al., 2019). The brain requires high amounts of energy for numerous processes, including neurotransmitter production and synaptic activity. Thus, the brain utilizes about 20% of the body’s total ATP, although it weighs only 2% of the entire body (Jain et al., 2010). Since mitochondrial energy demand is higher in the brain compared to other tissues, subtle changes in mitochondrial energy production have a strong impact on the brain (Joshi and Mochly-Rosen, 2018). In particular, the impaired neurotransmission and cognitive failure associated to neurodegenerative diseases may be related to the dysfunctional synaptic mitochondria that do not satisfy the high energy demands required at the synapses (Reddy and Beal, 2008). At pre-synaptic terminals, mitochondria produce most of the ATP required for exocytosis and neurotransmitter reuptake into synaptic vesicles, while at postsynaptic level, a high amount of energy is required mainly for plasma membrane excitability and receptor and ion channel functioning (Lores-Arnaiz et al., 2016). To study the bioenergetics and mitochondrial functions in the synaptic regions, synaptosomal fraction from the mammalian brain is a very useful tool. Here, we evaluate the mitochondrial function and efficiency, oxidative stress, inflammation, and BDNF pathway both in the brain cortex and in the corresponding synaptosomal fraction of HFD-induced obese mice.

## Materials and Methods

### Materials

The analytical-grade chemicals used were purchased from Sigma (St. Louis, MO, USA).

### Animal and Diet

Male C57Bl/6J mice (Charles River Laboratories, Calco, Lecco, Italy) were caged in a temperature-controlled room and exposed to daily 12 h light-12 h dark cycle with free access to water and food. Young animals (average 27 ± 0.8 *g*) were used. A group (*n* = 7) was sacrificed at the beginning of the study to establish baseline measurements. The remaining mice were divided into two experimental groups (*n* = 7 each): the first group received a standard diet (control diet, CD; 10.6% fat J/J; Mucedola 4RF21; Settimo Milanese, Milan, Italy) for 18 weeks; the second group received the HFD (50% fat J/J; Teklad# 93075) for 18 weeks. The last week of the treatment, the respiratory quotients (RQs) were measured in live animals using indirect open-circuit calorimeter. At the end of the experimental period, the animals were anesthetized by injection of chloral hydrate (40 mg/100 *g* body weight) and were killed by decapitation. Blood was taken from the inferior cava and serum was obtained by centrifuging at 1,000× *g* for 10 min and stored at −80°C for subsequent biochemical analyses. The cerebral cortex was removed and subdivided; samples not immediately used for synaptosomes and mitochondria preparation were frozen and stored at −80°C for subsequent determinations.

### Measurement of Oxygen Consumption, Carbon Dioxide Production, and Respiratory Quotient

Following an adaption period to the experimental environment, oxygen consumption (VO_2_) and carbon dioxide production (VCO_2_) were recorded by a monitoring system (Panlab s.r.l., Cornella, Barcelona, Spain) that is composed of a four-chambered indirect open-circuit calorimeter, designed for continuous and simultaneous monitoring. VO_2_ and VCO_2_ were measured every 15 min (for 3 min) in each chamber for a total of 6 h. The mean VO_2_, VCO_2_ and RQ values were calculated by the “Metabolism H” software (Dominguez et al., 2009).

### Body Composition and Energy Balance

During treatments, body weight and food intake were monitored daily to obtain body weight gain and gross energy intake. Energy balance assessments were conducted during the 18 weeks of feeding by comparative carcass evaluation (Mollica et al., 2017). The gross energy density for CD or HFD (15.8 or 21.9 kJ/g, respectively) and the energy density of the feces and the carcasses were determined by bomb calorimetric (Parr adiabatic calorimetric; Parr Instrument Company, Moline, IL, USA). Metabolizable energy (ME) intake was determined by subtracting the energy measured in feces and urine from the gross energy intake, which was determined from the daily food consumption and gross energy density. Evaluation of the energy, fat, and protein content in animal carcasses was conducted according to a published protocol (Mollica et al., 2017). Energy efficiency was calculated as the percentage of body energy gain per ME intake, and energy expenditure was determined as the difference between ME intake and body energy gain. Body energy gain was calculated as the difference between the body energy content at the end of the treatment and the energy content of the mice sacrificed at the beginning of the experiment (baseline measurements).

### Serum Parameters

The serum levels of triglycerides and cholesterol were measured by the colorimetric enzymatic method using commercial kits (SGM Italia, Rome, Italy, and Randox Laboratories Limited, Crumlin, UK). Glucose levels were determined by glucometer (Contour next, Ascensia, Switzerland). The serum levels of interleukin (IL)-1β, tumor necrosis factor-α (TNF-α; BioVendor, Brno, Czechia), insulin (Mercodia AB, Uppsala, Sweden), adiponectin and leptin (B-Bridge International, Mountain View, CA, USA) were measured using commercially available kits. As an index of insulin resistance (IR), HOmeostasis Model Assessment (HOMA)-IR was calculated using formula [HOMA = fasting glucose (mmol/L^−1^) × fasting insulin (μU/mL^−1^)/22.5].

### Brain and Synaptosomes Parameters

To determine the lipid peroxidation in cerebral cortex homogenate and synaptosomal fraction, the level of malondialdehyde (MDA) was measured using the thiobarbituric acid (TBAR) method. MDA reacts with thiobarbituric acid (TBA) to form a pink chromogen that is detected at the wavelength of 532 nm. MDA values were expressed as micromoles per milligram of protein (Lu et al., 2009). The levels of TNF-α and IL-1β in the cerebral cortex homogenate and in synaptosomal fraction were determined as previously reported (Cavaliere et al., 2018). Reduced GSH and oxidized glutathione (GSSG) concentration in the cerebral cortex homogenate and in the synaptosomal fraction were measured with the dithionitrobenzoic acid-GSSG reductase recycling assay; the GSH to GSSG ratio was used as an oxidative stress marker (Viggiano et al., 2016).

### Preparation of Mitochondria and Synaptosomes From the Cerebral Cortex

Synaptosomes were prepared using the standard procedure (Rao and Steward, 1991) yielding a well-characterized synaptosomal fraction (Eyman et al., 2007, 2013; Ferrara et al., 2009; Cefaliello et al., 2014; Penna et al., 2019). In brief, the cerebral cortex was quickly dissected and homogenized in a Dounce homogenizer with nine volumes of cold isotonic medium (HM) containing 0.32 M sucrose and 10 mM Tris-Cl, pH 7.4. To prepare subcellular fractions, following centrifugation of the homogenate (2,200 *g*, 1 min, 4°C), the sediment was resuspended in the same volume of HM and centrifuged under the same conditions to yield a sediment containing nuclei, cell debris and large synaptosomes (P1). The mixed supernatant fractions were centrifuged at higher speed (23,000 *g*, 4 min, 4°C) to yield a sediment that was resuspended in HM and centrifuged under the same conditions to yield a second sediment containing free mitochondria, synaptosomes and myelin fragments (P2) that was resuspended in HM. Differential centrifugation of P2 aliquots was used to prepare isolated synaptosomes and mitochondria. Further purification of synaptosomes was achieved by fractionating an aliquot of the P2 fraction on a discontinuous Ficoll gradient. One milliliter of the P2 fraction, brought to a final protein concentration of 3.5 mg/ml, was layered over a discontinuous gradient of 5% and 13% Ficoll dissolved in HM (2 ml each), and centrifuged at 45,000 *g*, for 45 min, 4°C. The purified synaptosomal fraction was recovered at the interface between the two Ficoll layers, diluted with nine volumes HM, and sedimented by centrifugation (23,000 *g*, 20 min, 4°C). The sediment was homogenized in HM and protein concentration was determined by Bradford colorimetric assay (Biorad) using bovine serum albumin (BSA) as standard. To obtain mitochondrial fraction the P2 fraction was resuspended in a medium containing 80 mM LiCl, 50 mM HEPES, 5 mM Tris-PO_4_, 1 mM EGTA and 0.1% (w/v) fatty-acid-free BSA, pH 7.0, and centrifuged at 500 *g*, 10 min, 4°C. The supernatant was centrifuged at 10,000 *g* for 10 min at 4°C, the pellet was washed once and resuspended in a medium containing 80 mM LiCl, 50 mM HEPES, 5 mM Tris-PO_4_, 1 mM EGTA and 0.1% (w/v) fatty-acid-free BSA, pH 7.0. The protein content of the mitochondrial fraction was determined by Bradford colorimetric assay (Biorad) using BSA as standard. The quality of isolated mitochondria was assured by checking that contamination of mitochondria by other ATPase-containing membranes was lower than 10%, and that addition of cytochrome c (3 nmol/mg protein) enhanced only state 3 respiration by approximately 10% (Cavaliere et al., 2016).

### Measurements of Mitochondrial Oxidative Capacities and Degree of Coupling

Oxygen consumption in isolated mitochondria was measured with high-resolution respirometry Hansatech oxygraph (Yellow Spring Instruments, Yellow Springs, OH, USA) at a temperature of 30°C. Isolated mitochondria were incubated in a medium (pH 7.0) containing 80 mM KCl, 50 mM HEPES, 5 mM KH_2_PO_4_, 1 mM EGTA and 0.1% (w/v) fatty-acid-free BSA to oxidize their endogenous substrates for a few minutes. Substrates were then added at the following concentrations: 10 mM succinate plus 3.75 mM rotenone; 10 mM pyruvate plus 2.5 mM malate. State 4 oxygen consumption was obtained in the absence of ADP, and State 3 oxygen consumption was measured in the presence of 0.3 mM ADP. The respiratory control ratio (RCR) was calculated as the ratio between states 3 and 4 according to Estabrook (1967). The degree of coupling was determined in the brain by applying equation by Cairns et al. (1998):

$$\text{degree of coupling=}\sqrt{\text{1}-{\left(\text{Jo}\right)}_{\text{sh}}\text{/}{\left(\text{Jo}\right)}_{\text{unc}}}$$

where (Jo)sh represents the oxygen consumption rate (OCR) in the presence of oligomycin that inhibits ATP synthase, and (Jo)unc is the uncoupled rate of oxygen consumption induced by carbonyl cyanide-p-trifluoromethoxyphenylhydrazone (FCCP), which dissipates the transmitochondrial proton gradient. (Jo)sh and (Jo)unc were measured as above using succinate (10 mmol/L) and rotenone (3.75 μmol/L) in the presence of oligomycin (2 μg/ml) or FCCP (1 μmol/L), respectively. Aconitase and superoxide dismutase (SOD) specific activity were measured spectrophotometrically as previously reported (Flohè and Otting, 1984; Cavaliere et al., 2016).

### Seahorse XFp Analyzer Cell Mito Stress Test

Oxygen consumption (OCR) and extracellular acidification rate (ECAR) measurements in synaptosomal fraction from mice cortex were performed by Seahorse XFp (Seahorse Biosciences, North Billerica, MA, USA), by using Cell Mito Stress Test kit (Seahorse Bioscience, 101706-100). XFp cartridges (Seahorse Bioscience) were hydrated by incubation with 200 μl calibrant (Seahorse Bioscience) per well at 37°C overnight. Before loading a cartridge onto the XFp analyzer, 20 μl of 30 μM oligomycin, 22 μl of 40 μM FCCP, 24 μl of 20 μM rotenone/antimycin A were added onto the cartridges (final concentrations of oligomycin, FCCP, and rotenone injected during the assay were 3 μM, 4 μM, and 2.0 μM, respectively). The cartridge was loaded onto the XFp analyzer for calibration. In each well of Seahorse XFp plate (Seahorse Bioscience, North Bilerica, MA, USA), precoated with poly-D-lysine, we seeded 10 μg of synaptosomal protein in a final volume of 100 μl ionic medium (20 mM HEPES, 10 mM D-Glucose, 1.2 mM Na_2_HPO_4_, 1 mM MgCl_2_, 5 mM NaHCO_3_, 5 mM KCl, 140 mM NaCl, pH 7.4 at 4°C). The plate was centrifuged at 2,000 *g* for 1 h at 4°C in swinging bucket rotor (Thermo Scientific 75003624) in a Thermo Fisher Scientific 3000R centrifuge. Before cell mito stress analyses, the medium was replaced with 180 μl of incubation medium (3.5 mM KCl, 120 mM NaCl, 1.3 mM CaCl_2_, 0.4 mM KH_2_PO_4_, 1.2 mM Na_2_SO_4_, 2 mM MgSO_4_, 4 mg/ml BSA, 15 mM D-glucose, 5 mM pyruvate, 2.5 mM malate, pH 7.4 at 37°C). The basal OCR was determined in the presence of the incubation medium. The proton leak was determined after inhibition of mitochondrial ATP production by oligomycin, as an inhibitor of the F0 F1 ATPase. Furthermore, the measurement of the ATP production in the basal state was obtained from the decrease in respiration by inhibition of the ATP synthase with oligomycin. Afterward, the mitochondrial electron transport chain was stimulated maximally by the addition of the uncoupler FCCP. Finally, the extra-mitochondrial respiration was estimated after the addition of the antimycin A and rotenone inhibitors of the complexes III and I, respectively. Coupling efficiency is the proportion of the oxygen consumed to drive ATP synthesis compared with that driving proton leak and was calculated as the fraction of basal mitochondrial OCR used for ATP synthesis (ATP-linked OCR/basal OCR). Spare capacity is the capacity of the cell to respond to an energetic demand and was calculated as the difference between the maximal respiration and basal respiration.

### Western Blot Analysis

The cerebral cortex and synaptosomal fraction were homogenized in lysis buffer (20 mM MOPS pH 7.4, 2 mM EGTA pH 8, 5 mM EDTA pH 8, 30 mM NaF, 60 mM β-Glycerophosphate, 1 mM Sodium orthovanadate, 1% Triton X-100, 1 mM DTT) with a cocktail of protease inhibitors (Sigma-Aldrich). Western blot analyses were performed as previously described (Chun et al., 2004). Briefly, proteins (20 or 40 μg/lane) were separated on 12% SDS-PAGE and transferred to nitrocellulose membranes. The blots were incubated with anti-BDNF polyclonal antibody (Santa Cruz Biotechnology, 1:200), anti-phospho-CREB, and anti-CREB, mouse and rabbit monoclonal antibodies, respectively, anti-TrkB (80E3) rabbit monoclonal antibody (Cell Signaling Technology; 1:1,000), and anti-GAPDH rabbit antibody (Sigma-Aldrich; 1:8,000) overnight at 4°C, and then with secondary antibody against mouse or rabbit IgG (Sigma-Aldrich; 1:1,000–2,000) for 1 h at RT. The signals were visualized with the ECL system (Pierce). The same membrane was used to test all reported markers and the expression level of GAPDH was used to normalize the data.

### Statistical Analysis

All data are presented as means ± SEM. Differences among groups were compared by unpaired *t*-test. Differences were considered statistically significant at *p* < 0.05. All analyses were performed using GraphPad Prism (GraphPad Software, San Diego, CA, USA).

### Ethics Statement

This study was carried out in strict accordance with the Institutional Guidelines and complied with the Italian D.L. no. 116 of January 27, 1992, of Ministero Della Salute and associated guidelines in the European Communities Council Directive of November 24, 1986 (86/609/ECC). All animal procedures reported herein were approved by the Institutional Animal Care and Use Committee (CSV) of the University of Naples Federico II.

## Results

### Oxygen Consumption, Carbon Dioxide Production, and Respiratory Quotient

O_2_ consumption (ml/min/Kg^0.75^ bw) of HDF mice was not significantly different from control animals (15.82 ± 0.55 vs. 17.06 ± 0.46), while CO_2_ production (ml/min/Kg^0.75^ bw; 12.22 ± 0.58 vs. 15.69 ± 0.48; *p* < 0.05) and RQ (0.77 ± 0.02 vs. 0.92 ± 0.01; *p* < 0.05) were significantly decreased compared to control mice. RQ index reflects the ratio of carbohydrate/fatty acid oxidation, therefore the decreased RQ index in HFD-fed animals demonstrates that these animals used fatty acid as a preferential fuel source compared to control animals.

### Body Composition and Energy Balance

HFD consumption produced a significant increase in body weight, body lipids percentage, lipid gain, and body energy compared to the control diet. HFD mice exhibited a decrease in body water and body protein percentage compared to control mice, while protein gain did not change between groups. In addition, HFD animals exhibited a higher ME intake, energy expenditure and energy efficiency compared to control mice (Table 1). These data are in agreement with our previous results (Mollica et al., 2017).

**Table 1:** Body composition and energy balance in control and high-fat diet (HFD) mice.

	CD	HFD
Body weight gain, g	11.1 ± 1.56	18.65 ± 1.44*
Lipid, %	9.6 ± 0.8	31.9 ± 4.2*
Protein, %	21.6 ± 0.8	17.0 ± 1.1*
H_2_O, %	63.7 ± 1.96	46.0 ± 2.1*
Body energy content, kJ/g	8.45 ± 0.43	16.9 ± 1.91*
Body weight gain, kJ	94.0 ± 12.8	411.2 ± 50.7*
Metabolizable energy intake, kJ	4828.0 ± 152.6	5516.0 ± 163.8*
Energy expenditure, kJ	4734.0 ± 81.8	5104.8 ± 106.8*
Energy efficiency	0.02 ± 0.002	0.07 ± 0.008*
Protein gain, kJ	57.2 ± 6.6	66.1 ± 21.7
Lipid gain, kJ	59.0 ± 13.7	414.0 ± 50.4*

Data are presented as means ± SEM from n = 7 animals/group. *p < 0.05 compared to CD group. CD, control diet; HFD, high-fat diet.

### Serum Metabolites and Inflammatory Parameters

**Figure 1:**
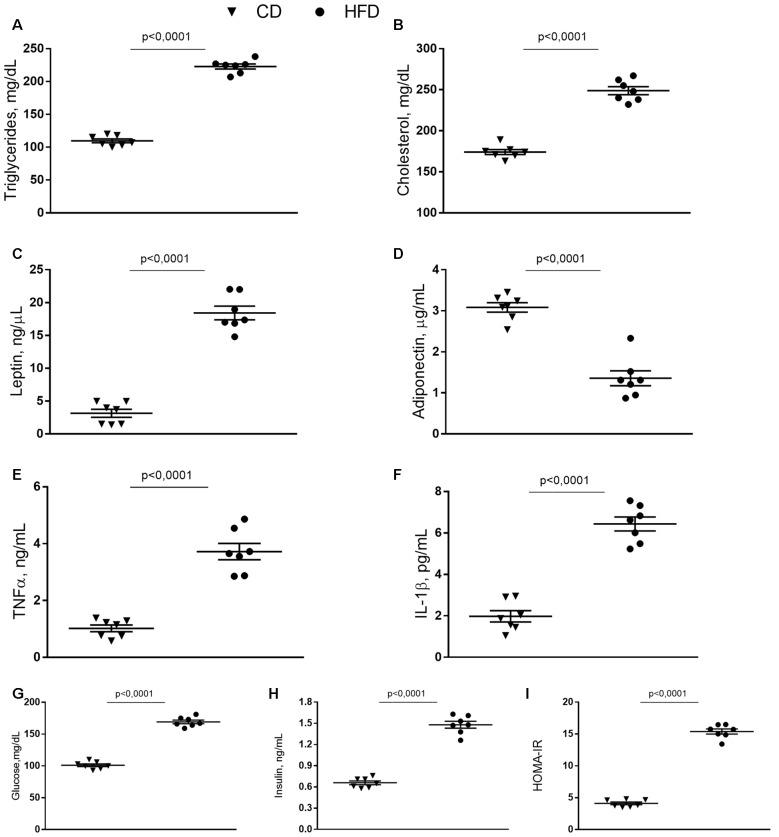
Effect of high-fat diet (HFD) on serum metabolic parameters and proinflammatory markers. Triglycerides **(A)**, cholesterol **(B)**, leptin **(C)**, adiponectin **(D)**, tumor necrosis factor α (TNF-α, **E**), interleukin 1β (IL-1β, **F**), glucose **(G)**, insulin **(H)** serum levels, and homeostasis model assessment of insulin resistance (HOMA-IR, **I)** are reported. Data are presented as means ± SEM from *n* = 7 animals/group. Triangle: control diet (CD), circle: high-fat diet (HFD). Differences have been evaluated by unpaired *t*-test.

HFD mice exhibited increased serum levels of triglycerides and cholesterol compared to control animals (Figures 1A,B), in agreement with our previous results (Mollica et al., 2017). Leptin concentration significantly increased in HFD mice compared to controls, while adiponectin concentration decreased in HFD animals compared to the control group (Figures 1C,D). Serum levels of TNF-α, and IL-1β, significantly increased in the HFD group compared to controls (Figures 1E,F). Compared with the control group, HFD mice showed a marked increase in glucose and insulin level and HOMA-IR (Figures 1G–I).

### Inflammation and Oxidative Stress in Brain Cortex

**Figure 2:**
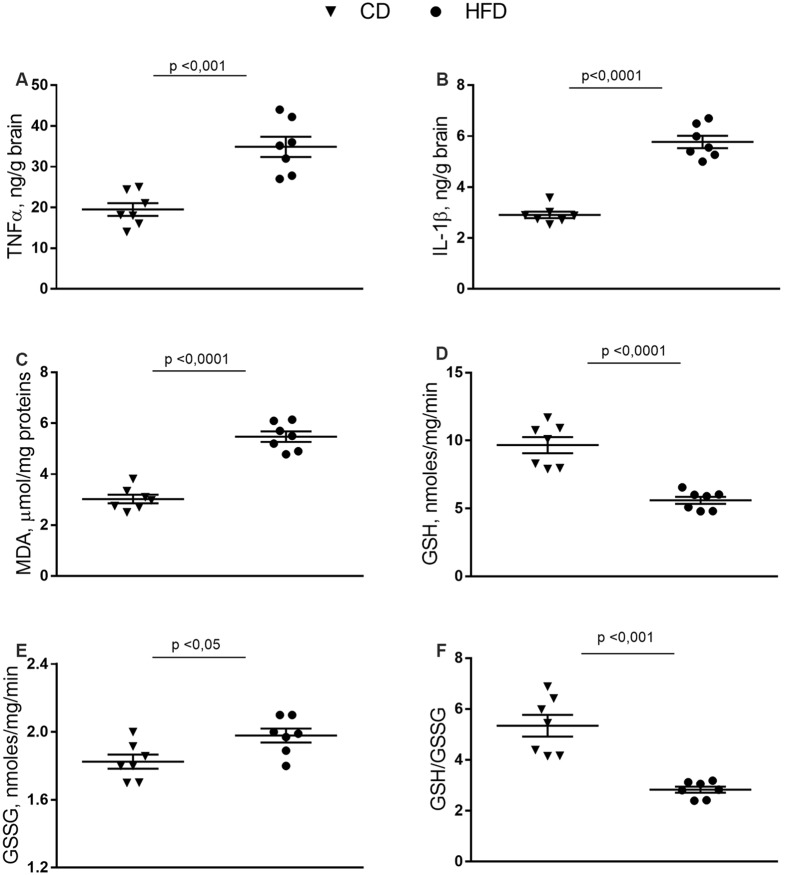
Effect of HFD on redox status and pro-inflammatory parameters in cerebral cortex tissue. Tissue levels of TNF-α **(A)**, IL-1β **(B)**, malondialdehyde (MDA, **C**), glutathione (GSH, **D**), oxidized glutathione (GSSG, **E**) and GSH/GSSG ratio **(F)** are reported. Data are presented as means ± SEM from *n* = 7 animals/group. Triangle: control diet (CD), circle: high-fat diet (HFD). Differences have been evaluated by unpaired *t*-test.

In the brain cortex of HFD group TNF-α, IL-1β, and MDA content was significantly higher than control mice (Figures 2A–C). GSH content significantly decreased in the HFD animals compared to controls (Figure 2D), while no difference was observed in GSSG content between groups (Figure 2E). Consistently, the HFD mice exhibited a lower GSH/GSSG ratio compared to controls (Figure 2F), indicating that HFD affected the oxidant state of the brain cortex.

### Inflammation and Oxidative Stress in Synaptosomes From Brain Cortex

**Figure 3:**
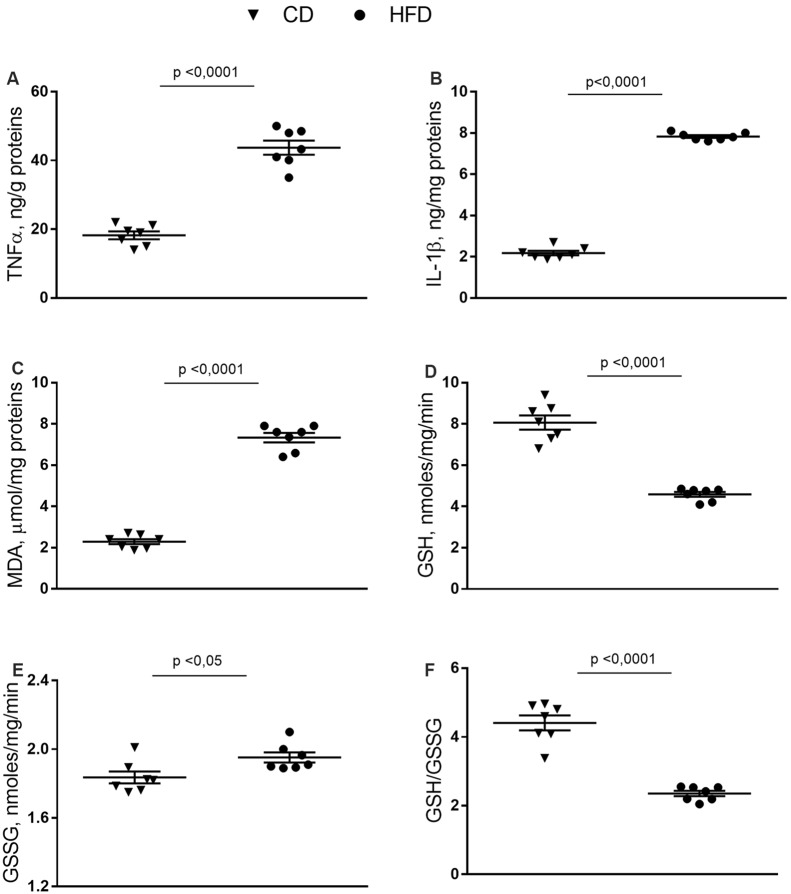
Effect of HFD on redox status and pro-inflammatory parameters in synaptosomal fraction. Synaptosomal levels of TNF-α **(A)**, IL-1β **(B)**, MDA **(C)**, GSH **(D)**, GSSG **(E)** and GSH/GSSG ratio **(F)** are reported. Data are presented as means ± SEM from *n* = 7 animals/group. Triangle: control diet (CD), circle: high-fat diet (HFD). Differences have been evaluated by unpaired *t*-test.

In synaptosomes from the HFD group, the inflammatory state was more pronounced than in the brain cortex homogenate. Indeed, the TNF-α, IL-1β and MDA content were about 140%, 240%, and 220% higher than control mice (Figures 3A–C). GSH content significantly decreased in HFD animals compared to controls (Figure 3D), while no difference was observed in GSSG content between groups (Figure 3E). Moreover, HFD mice exhibited a lower GSH/GSSG ratio compared to controls (Figure 3F).

### Mitochondrial Function, Efficiency and Oxidative Stress in Brain Cortex

**Figure 4:**
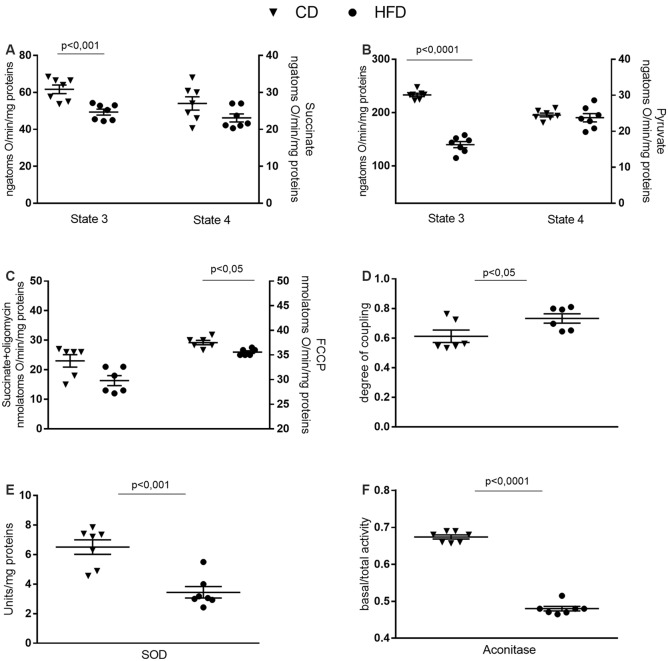
Effect of HFD on mitochondrial respiration parameters in the cerebral cortex. Mitochondrial respiration rates measured in the presence of succinate **(A)**, pyruvate **(B)** as substrates, oxygen consumption in the presence of oligomycin or uncoupled by carbonyl cyanide 4-(trifluoromethoxy) phenylhydrazone (FCCP, **C**), degree of coupling **(D)**, superoxide dismutase (SOD, **E**) and aconitase activities **(F)** are reported. Data are presented as means ± SEM from *n* = 6 or 7 animals/group. Triangle: control diet (CD), circle: high-fat diet (HFD). Differences have been evaluated by unpaired *t*-test.

Mitochondrial state 3 respiration, evaluated using succinate or pyruvate as substrates, significantly decreased in the HFD mice compared to control, while no variation in state 4 was detected between groups (Figures 4A,B). Oligomycin state 4 respiration showed a trend of reduction in the HFD mice, and maximal FCCP-stimulated respiration was significantly reduced in in the HFD mice compared to control (Figure 4C). Consequently, brain mitochondria energetic efficiency, evaluated as degree of coupling, was increased in the HFD mice (Figure 4D). SOD and aconitase activities were significantly lower in HFD than in control mice (Figures 4E,F).

### Mitochondrial Function in the Synaptosomal Fraction

The mitochondrial function of the synaptosomal fraction was examined using the Seahorse XFp cell mito stress kit (Seahorse Bioscience, North Billerica, MA, USA). It was performed in real-time at basal level and following a sequential addition of mitochondrial respiration inhibitors: oligomycin, FCCP, and a combination of antimycin A and rotenone. Oligomycin, an inhibitor of ATP synthase, was used to distinguish between oxygen consumption that cells use to synthesize ATP (ATP-linked respiration) and oxygen consumption that is used to overcome the proton leak across the mitochondrial membrane. FCCP treatment collapses the proton gradient and disrupts the mitochondrial membrane potential, which allows measurement of the maximal uncoupled respiration (maximal respiration). A combination treatment of rotenone, a complex I inhibitor, and antimycin A, a complex III inhibitor, was used to shut down mitochondrial respiration, which enables differentiation between the mitochondrial (basal respiration) and non-mitochondrial respiration contribution to total respiration. The difference between maximal and basal respiration constitutes the spare capacity.

**Figure 5:**
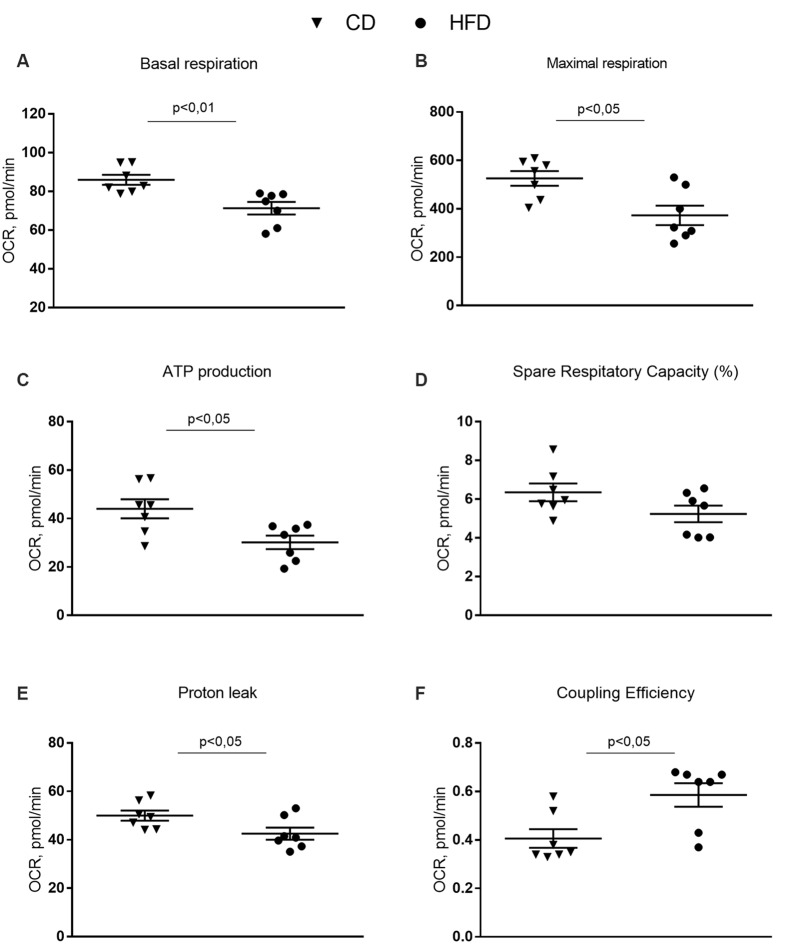
Effect of HFD on mitochondrial respiration parameters in synaptosomal fraction. Basal respiration **(A)**, maximal respiration **(B)**, ATP production **(C)**, spare respiratory capacity percentage **(D)**, proton leak **(E)** and coupling efficiency **(F)** are reported. Data are presented as means ± SEM from *n* = 7 animals/group. Triangle: control diet (CD), circle: high-fat diet (HFD). Differences have been evaluated by unpaired *t*-test.

The results from the Cell Mito Stress Test on synaptosomal fractions showed a decrease in the basal respiration in the HFD mice compared to controls (Figure 5A). These results are consistent with the decreased maximal rate of respiration and ATP production in HFD animals compared to controls (Figures 5B,C). Spare respiratory capacity, i.e., the capacity of the cells to respond to an energetic demand by generating ATP *via* oxidative phosphorylation (OXPHOS), was not different between groups (Figure 5D). Proton leak significantly decreased and, as a consequence, coupling efficiency increased in the HFD mice compared to control (Figures 5E,F).

### Effect of HFD on the BDNF Pathway in Brain Cortex and Synaptosomal Fraction

**Figure 6:**
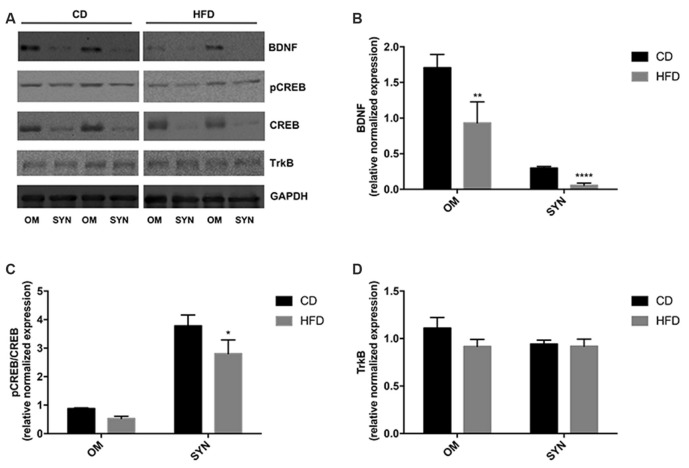
Effect of HFD on the BDNF pathway in the brain cortex homogenate and synaptosomal fraction. Expression levels of BDNF **(B)**, pCREB/CREB **(C)**, and TrkB **(D)** were normalized with that of GADPH in the brain cortex homogenate (OM) and synaptosomes (SYN) from control diet (CD) and high-fat diet (HFD) group. Panel **(A)** shows representative immunoreactive signals. Data are presented as means ± SEM from *n* = 4 animals/group. *p < 0.05, **p < 0.01, and ****p < 0.0001 compared to control mice. CD, control diet; HFD, high-fat diet.

The modulation of the BDNF pathway by HFD was evaluated by western blot analysis. We demonstrated that in both brain cortex and synaptosomal fraction from HFD mice the expression level of BDNF was significantly decreased compared to CD animals. Notably, the decrease was more pronounced in the synaptosomal fraction than in the homogenate (80% vs. 50%; Figures 6A,B). In addition, we also observed that the HFD decreased the CREB phosphorylation level both in homogenate and synaptosomes, although the effect was significant only in synaptosomal fraction (Figures 6A–C). No difference was observed in the TrkB expression level between the HFD and the CD group (Figures 6A–D).

## Discussion

The main finding of this study is that HFD consumption in mouse has a detrimental influence on brain cortex bioenergetic, altering mitochondrial function, efficiency and oxidative stress. Notably, these alterations were observed also in mitochondria from synaptosomal fractions. It is noteworthy that the dysfunctions of presynaptic mitochondria may be one of the underlying mechanisms contributing to cognitive decline in neurodegenerative diseases (Devine and Kittler, 2018). As known, HFD increases ME intake, metabolic efficiency, weight gain, and body lipid levels, leading to metabolic alterations, such as dyslipidemia, associated with low-grade inflammation. The effects on body weight and lipids can be explained by a decrease in energy expenditure and an increase in energy efficiency. The impaired ability to use fat as a metabolic fuel of HFD animals, suggests that the large part of the higher energy intake is stored as fat, confirming previous reports (Mollica et al., 2017; Cavaliere et al., 2018). In addition, in HFD the discrepancy between fatty acid uptake and utilization leads to the accumulation of toxic lipid species resulting in overproduction of ROS, IR, and inflammation. Our data, showing dysregulation of leptin and adiponectin serum levels in the HFD animals, confirm the link between these adipokines, obesity-related metabolic disorders, and inflammation. In particular, decreased adiponectin levels in the HFD group is associated with increased serum levels of TNF-α and oxidative stress (Chakraborti, 2015). Interestingly, we observed increased levels of TNF-α and oxidative stress also in brain cortex of the HFD mice, confirming that neuroinflammation observed in the diet-induced obesity animal model may be linked to increase in TNF-α secretion. It is noteworthy that the inflammation and oxidative stress is even more pronounced in the synaptosomal fraction from the brain cortex. The synaptosomes are vescicles produced *in vitro*, during homogenization of nervous tissue, that contain all the components of synaptic regions *in vivo* (Eyman et al., 2007; Crispino et al., 2009, 2014). Therefore, the synaptosomal fraction is a good model to study the modulation occurring at the synaptic level. Neuronal plasticity reflects the ability of synapses to be modified in number and strength in response to physiological and pathological stimuli. As a consequence, analyzing the dynamics of the synaptic region of brain areas opened a new perspective in the investigation of the molecular mechanisms underlying the physiological and pathological responses of the nervous system. Our data, indicating that inflammation and oxidative stress in the synaptic area is even higher than in the brain cortex homogenate, are particularly relevant; indeed, since impaired synaptic plasticity has been associated to neurodegeneration (Merlo et al., 2016), the HFD-induced inflammation may be indicated as a possible risk factor for the development of these diseases. Interestingly, recent data showed that acetylcholine plays an anti-inflammatory role in inhibiting NF-kB activity and suppressed the production of TNF-α and other proinflammatory cytokines (Chavan et al., 2017). We also demonstrated that the altered inflammatory state of the HFD animals can be linked to mitochondria dysfunctions. In the central nervous system, mitochondria have a crucial role in controlling energy homeostasis, which is essential for cognitive functions. In particular, mitochondria specifically located at synapses play a key role in providing energy to support synaptic functions and plasticity, thus their defects may lead to the synaptic failure responsible for neurodegenerative disease (Reddy and Beal, 2008). Therefore, we analyzed the effect of HFD on mitochondria isolated from the brain cortex and from cerebral cortex synaptosomes. Our data demonstrated that HFD induced alterations in the brain cortex and synaptic mitochondria. Mitochondria from the brain cortex of HFD mice exhibited a reduced respiratory capacity. Indeed, state 3 respiration significantly decreased both in presence of NADH-linked (pyruvate) and FADH-linked (succinate) substrate. A decrease in succinate State 3 respiration may be due to defects in the activity of substrate oxidation reactions (complex II, complex III, complex IV, and dicarboxylate carrier) and/or in the activity of the phosphorylation reactions (ANT, ATP synthase and phosphate carrier). In the presence of succinate plus FCCP, we also observed a decreased oxygen consumption in the HFD mice. These results suggest that the HFD-induced impairment of mitochondrial functions is not related to phosphorylating reactions but depends on altered substrate oxidation reactions. Indeed, when respiration is measured in the presence of succinate plus FCCP, the phosphorylation reactions do not exert control over respiration. Moreover, HFD mice showed an increased degree of coupling and, as a consequence, an increased mitochondrial efficiency. Mitochondria generate ATP by oxidizing nutrients, and the energy generated by the electron transport is utilized to phosphorylate ADP to ATP. Electron transport and ATP synthesis are tightly coupled, but some of the energy generated by electron transport is uncoupled from ATP synthesis. This mitochondrial uncoupling dissipates part of the proton gradient across the inner membrane without generating ATP (Nedergaard et al., 2005; Tseng et al., 2010). Increased mitochondrial energy efficiency implies that less substrate needs to be burned to obtain the same amount of ATP. As a consequence, the excess of unburned substrate favors lipid deposition. The partial block of electron flow within the respiratory chain, due to respiratory chain impairment, and the decrease in mitochondrial uncoupling both contribute to the enhanced oxidative stress (MDA, aconitase activity) observed in the HFD mice. In fact, the uncoupling is a major mechanism for the adjustment of the membrane potential to control mitochondrial ROS emission. By mildly uncoupling, the mitochondria can avoid the oversupply of electrons/reducing equivalents into the respiratory complexes and minimize the likelihood of electron interaction with oxygen (Skulachev, 1998). In addition, the HFD animals also showed a decrease in SOD enzyme activity, the first line of defense against oxidative stress (Lu et al., 2009), contributing to the increased oxidative stress of these animals. Accordingly, HFD induces an imbalance of ratio GSH/GSSG, a clear sign of altered redox state in cells. HFD also induced impairment of synaptic mitochondrial functions. Indeed, in the HFD mice, we observed a reduced basal and maximal respiration, and a reduced ATP production. Moreover, these animals also showed an increased coupling efficiency linked to a decreased proton leak. These features can be responsible for the increased oxidative stress and the inflammatory state that was observed in synaptic regions of the HFD mice. Since neuroinflammation is known to affect BDNF signaling in the brain (Lima Giacobbo et al., 2019), we investigate the BDNF and CREB expression levels in brain cortex and synaptosomal fraction from the same region. BDNF is a neurotrophin that plays an important role in plasticity of the central nervous system and is also involved in the pathogenesis of neurological diseases (Autry and Monteggia, 2012). In particular, BDNF has a short-term effect in synaptic plasticity influencing post-translation modification of proteins already available at the synapse, but also long-term effects including the modulation of the synaptic system of protein synthesis (Leal et al., 2014). Our results indicate a significant decrease in BDNF expression in both HFD homogenate and synaptosomal fractions. Therefore, it is possible to hypothesize that, in HFD animals, the decreased synaptosomal level of BDNF results in a decrease of the synaptic protein synthesis, affecting in turn mitochondria activity. Indeed, recent data demonstrated that axonal protein synthesis is crucial for local mitochondrial maintenance, and its alteration may lead to neuropathologies (Cioni et al., 2019). It has been also shown that the mitochondria located at the synaptic level fuel the local protein synthesis necessary for synaptic plasticity (Rangaraju et al., 2019). Thus, this two-way crosstalk between local translation and mitochondria function in synapse may represent an innovative mechanism to guide neuronal plasticity (Rossoll and Bassell, 2019). The modulation of BDNF that we observed in the HFD animals was accompanied by a decreased phosphorylation of its upstream factor CREB in the synaptosomal fraction, confirming that HFD has a relevant effect on molecular plasticity pathways located at the synaptic level. Moreover, BDNF in the brain is one of the key proteins in food intake regulation and body weight control (Lapchak and Hefti, 1992), playing a critical role in regulating feeding and energy balance (Noble et al., 2011; Rios, 2013). BDNF inversely correlates with fasting plasma glucose and homeostasis model assessment of insulin resistance score (HOMA-IR), which is an indirect measure of IR (Eyileten et al., 2017). In line with these findings, our results related to altered food intake, energy balance, body weight and HOMA index of HFD group correlate with the reduced levels of BDNF that we observed in the same group of animals. We performed our experiments selectively on male animals that are free from estrous-related hormonal changes affecting brain physiology. Nonetheless, it will be of interest to extend these analyses to female animals to investigate a possible gender difference in the response of the brain to HFD.

## Conclusion

Our data provided evidence regarding the link between HFD and low-grade systemic inflammation, but also inflammation and oxidative stress in the brain cortex. The effect is even more pronounced in the synaptic regions indicating the strong impact of the diet on neuronal plasticity. The oxidative stress depends on the overproduction of free radicals that is partially due to the impaired mitochondrial functions. Indeed, HFD induces in brain mitochondria a decrease in mild uncoupling, that has the role to maintain mitochondrial membrane potential below the critical threshold for ROS production. Therefore, our data indicate that improving mitochondria functions may be used as a strategy to counteracts neuroinflammation and brain oxidative stress.

## Data Availability Statement

The datasets generated for this study are available on request to the corresponding author.

## Ethics Statement

The animal study was reviewed and approved by Institutional Animal Care and Use Committee (CSV) of University of Naples Federico II.

## Author Contributions

MPM and MC conceived and designed the experiments, analyzed the data, and wrote the manuscript. GC, GT, EP, FC, CP, AL, CA, and AC performed the experiments, collected the data, and performed the data analyses. GMR, RM, MM, GM, and CZ contributed to the discussion and to the editing of the manuscript. MC was the guarantor of this work and, as such, had full access to all the data in the study and takes responsibility for the integrity of the data and the accuracy of the data analysis.

## Conflict of Interest

The authors declare that the research was conducted in the absence of any commercial or financial relationships that could be construed as a potential conflict of interest.
